# Estimating the Size of Populations at High Risk of Malaria in 2 Operational Districts in Cambodia: Household-Based Survey

**DOI:** 10.2196/58584

**Published:** 2024-09-27

**Authors:** Jerry O Jacobson, Dyna Doum, Neil F Lobo, Siv Sovannaroth, Allison Tatarsky, David J McIver

**Affiliations:** 1 Malaria Elimination Initiative Institute for Global Health Sciences University of California, San Francisco San Francisco, CA United States; 2 Health Forefront Organization Phnom Penh Cambodia; 3 Eck Institute for Global Health University of Notre Dame South Bend, IN United States; 4 National Center for Parasitology, Entomology and Malaria Control Phnom Penh Cambodia

**Keywords:** population size estimate, malaria, forest exposure, Greater Mekong Subregion, infectious, epidemiology, epidemiological, size, population, communicable, risks, surveys, questionnaires

## Abstract

**Background:**

Cambodia is targeting the elimination of malaria by 2025. The last remaining pockets of malaria in Cambodia are concentrated among populations exposed to forested areas, but the size of these populations is not well understood. To plan for the procurement and distribution of vector-control tools, chemoprophylaxis, and other commodities for malaria prevention and surveillance, robust estimates of the population at greatest risk are required.

**Objective:**

This study aims to estimate the number of forest-exposed individuals residing in Cambodia’s highest-burden operational districts (ODs) in 2 provinces with active malaria transmission.

**Methods:**

In April 2023, a multistage, in-person survey was conducted among residents in the 2 ODs in Cambodia with the highest malaria burden: Sen Monorom in Mondulkiri province and Phnom Srouch in Kampong Speu province. In each OD, 10 villages were randomly selected, and 35 households were randomly selected from each village. To estimate the number of individuals at high risk of malaria—defined as residing within 1 km of a forest or traveling at least once per week to the forest—respondents were asked about the distance from their household to the nearest forested area, and their travel patterns to forested areas. To account for mobility (ie, to avoid double-counting), respondents also provided information on overnight stays at other households in the selected villages in the past month. In the 4 selected villages in Sen Monorom OD where Project BITE forest packs (an intervention in the larger research program) had been distributed prior to the survey, respondents were also asked questions to determine if they had received such a pack, to develop smaller scale “multiplier method” estimates of at-risk individuals in each of those villages.

**Results:**

In Sen Monorom, 138 households and 872 individuals were enrolled in the survey, and in Phnom Srouch, 163 households and 844 individuals were enrolled. The estimated percentage of female householders was 49.7% (852/1716) across both ODs; the median age was 22 (IQR 12-37) years in Sen Monorom and 24.5 (IQR 16.0-40.5) years in Phnom Srouch (total age range 3-86). Based on mobility-adjusted survey estimates alone, 32% (280/706; 95% CI 19.9-47.2) of residents in Sen Monorom (an estimated 12,133-20,135 individuals) and 36% (68/198; 95% CI 24.5-45.5) of residents in Phnom Srouch (an estimated 1717-2203 individuals), met risk criteria for forest exposure. Between 125 and 186 individuals were estimated to be at risk in each of the 4 villages where the multiplier method could be applied.

**Conclusions:**

This study provides estimates of the number of individuals potentially at high risk for malaria infection due to forest exposure in 2 ODs in Cambodia. These estimates can support planning for malaria control and elimination efforts. The straightforward methods of household surveys and multipliers should be feasible for many national malaria control programs.

## Introduction

Accurate estimates of the number of individuals at high risk for malaria are essential for national malaria programs to plan and implement prevention activities, forecast and procure commodities, to assess and monitor program coverage, advocate for resources, and set targets for burden reduction and elimination. Yet, the population subgroups that remain at high risk for malaria in countries that have made significant progress toward malaria elimination are often not assessed or monitored by existing programs or population censuses.

Malaria continues to be an important cause of morbidity and mortality in the Greater Mekong Subregion (GMS). While the number of cases of malaria in the GMS has decreased by 77%, and the number of deaths decreased by 97%, between 2012 and 2022 [1], the number of indigenous cases rose from 90,082 in 2021 to 170,527 in 2022 [2]. In Cambodia, as throughout the GMS, the 2 groups at increased risk for malaria include the forest dwellers (who normally reside in the forest or on the forest fringe) and forest goers (who normally reside in nonforest residential areas and travel to the forest for a variety of reasons) [3,4]. Population size estimates for forest-exposed populations are generally unavailable in Cambodia.

This study aims to apply a simple and replicable method—a household survey—to estimate the number of people at risk of malaria due to forest exposure in the 2 health operational districts (ODs) with the greatest annual parasite incidence within the 2 highest-burden provinces in Cambodia at the time of this study: Sen Monorom OD in Mondulkiri province and Phnom Srouch OD in Kampong Speu province. In 2021, these ODs had an annual parasite incidence of 5.7 and 2.5 cases per 1000 population, respectively, based on data from the malaria information system (MIS) of the Cambodia National Center for Parasitology, Entomology and Malaria Control. In addition, the study aimed to develop smaller area estimates for selected villages, by combining the household survey data with Project BITE (bite interruption toward elimination) implementation research study data, using the “multiplier method.” Project BITE is a large research program that include the distribution and evaluation of “forest packs” containing vector-control tools for at-risk populations in targeted ODs. Various refinements were introduced in the design of the survey to reduce potential error in the population size estimates due to the at-risk population’s frequent travel away from the household and possible underreporting of traveling to the forest due to the illicit nature of some forest activities.

## Methods

### Project BITE Program Data

Between October 2022 and January 2023, Project BITE, Cambodia National Center for Parasitology, Entomology and Malaria Control, and Malaria Consortium—an international nongovernmental organization supporting malaria elimination activities in Cambodia—distributed “forest packs” primarily via village malaria workers (VMWs) to forest dwellers and forest goers in Sen Monorom and Phnom Srouch (forest rangers were also targeted, but not included in this study). The packs contained mosquito bite prevention tools (topical repellent, volatile pyrethroid spatial repellent, and etofenprox treatment for clothing) and were distributed monthly, for 4 months, in villages that were active or recently active *Plasmodium falciparum* foci. In total, 14,000 packs were distributed in Sen Monorom OD and 6731 packs in Phnom Srouch OD to a total of 5744 individuals across both ODs. Individuals could choose to receive and use any or all of the items in the pack. Forest dwellers were defined as individuals who resided in a living structure inside the forest or within 1 km of the forest edge, while forest goers were defined as those traveling at least once per week to the forest while residing at least 1 km from the forest edge. At each distribution round, field teams aimed to provide packs to the same households and individuals; however, identities were not verified.

### Household Survey

Between April 3, 2023, and April 18, 2023, a household survey was conducted to estimate the number of forest dwellers and forest goers in the 2 selected ODs, in all villages with any *P falciparum* cases in 2022. In Phnom Srouch, all 10 villages with active foci were selected. In Sen Monorom, 10 of 38 villages (26%) with active foci were randomly selected by probability proportional to size without replacement, reaching the desired sample size [5], with size defined as the number of *P falciparum* cases per population. In each selected village, 35 households were selected by simple random sampling from a list of residential dwellings developed with the assistance of village leaders. A study team consisting of an interviewer and a VMW went to the selected households in randomized order until information had been gathered on the target sample size of 80 household members aged 3 years and older. In each household, the household head or other adult aged 18 years or older present, was asked to respond to a series of questions on behalf of all such individuals (henceforth, survey “participants” or “householders”), including all usual residents and any visitors who had slept at the household on the previous night, whether or not they were family members. The survey (Multimedia Appendix 1) was interviewer-administered at the household by tablet and queried demographic characteristics, patterns of sleeping away from the household in the past 4 weeks, and receipt of BITE tools. While the primary respondent was asked to complete the survey on behalf of all householders, any householder present could respond for themselves if they provided consent. If participants indicated that the household was 1 km or greater from the forest, additional questions were asked regarding travel to the forest to date during the year.

As confidentiality protections, GPS coordinates of households were not recorded, respondents were not asked about the nature of forest activities, and only initials—rather than names—were recorded on survey forms.

### Survey Sample Size Calculations

The survey sample size was determined based on a desired precision of +/– 5% for the estimated percentage of householders that belong to the target population in a given OD, based on a 25% baseline prevalence of belonging to the target population (based on rough data previously collected by Project BITE), a total OD population size of 50,000 eligible residents, a nonresponse rate of 10%, a design effect of 2.5 due to the multistage sampling design, and a 95% CI. These parameters led to a minimum sample of 796 residents per OD. Results were similar when assuming a total population of 25,000 eligible residents. Assuming an average of 3 individuals per household would meet age and other eligibility criteria led to a minimum of 265 households per OD; however, since there were no available data to check this assumption, the study team planned, conservatively, that it might be necessary to visit up to 30% more, or 345 households. Budget considerations allowed for sampling up to 10 villages per OD. Dividing the sample evenly across villages led to sample size requirements of 80 residents and 35 households per village. However, once the sample size of 80 residents was met, no further households were enrolled in the village. If the final household had more eligible residents than required to meet the target number of residents, data on all eligible residents were collected in the survey.

### Statistical Analysis

Participants who indicated that their household was located “inside the forest or within 1 km of the forest” were classified as “forest dwellers.” Where responses among householders differed (16 households), all householders were classified according to the majority response. Participants not classified as forest dwellers were classified as “forest goers” if they had traveled to the forest at least 1 day per week anytime during the dry season “so far this year, up to today.”

### Household-Based Size Estimates

The number of people at risk in each OD was calculated by multiplying the resident population of the OD by the survey-estimated proportion of residents who met the criteria as a forest dweller or forest goer. To develop a 95% CI, the limits of the CI of the proportion were multiplied by the resident population. The population counts for Sen Monorom OD were from the Cambodia National MIS. For Phnom Srouch OD, we obtained the population counts by calling all of the village heads, since the data in the MIS for Kampong Speu province were incomplete.

### Multiplier Size Estimates

We used the “multiplier method,” often used in HIV epidemiology [6,7], as a second size estimation strategy. The “multiplier method” draws on 2 data sources. One is a representative survey—like the household survey described here—and the second is a count of the number of people in a known subgroup of the target population (the “multiplier”). The multiplier in this study was the number of people who had been given a Project BITE forest pack, based on Project BITE program data. Multiplier-based size estimates could only be calculated for villages where forest packs had been distributed. In each such village, the multiplier size estimate was calculated as *M*/*p*, where *M* was the number of recipients of forest packs, approximated as one-fourth of the number of forest packs distributed in the village (whether complete or incomplete) over the 4 distribution rounds. The parameter *p* was the survey-estimated proportion of at-risk householders (whether forest dwellers or forest goers) who reported (1) receiving “any items to help prevent mosquito bites from a VMW as part of Project BITE” between October 2022 and January 2023 and (2) mentioned topical repellent, spatial repellent, or treated clothing, when asked in an open-ended question which items they received. The multiplier size estimates were calculated only for villages in Sen Monorom, due to data quality issues with the BITE forest pack distribution records for Phnom Srouch. CIs for the multiplier-based size estimates were calculated as in Fearon et al [8] to account for sampling error in *p*.

### Sampling Weights

Survey-estimated proportions were weighted, with sampling weights calculated as the inverse of the probability the participant’s household was selected as follows: P(participant selected)=P(village selected)P(household selected). In Sen Monorom, P(village selected) was the probability the village was selected by probability proportional to size, as calculated by the *Sampling* package in *R* statistical software (R Foundation for Statistical Computing), whereas in Phnom Srouch it was 1, as all villages with active foci were surveyed. P(household selected) was the sampling fraction of households: (number of households enrolled)/(number of households in village). The standardized sampling weights ranged from –0.7 to 2.6 in Sen Monorom and from –1.3 to 1.8 in Phnom Srouch.

### Mobility Adjustment

To reduce the likelihood of double-counting individuals who frequently spend the night at multiple households in the same OD—a common occurrence in these locations—we developed mobility correction factors for the OD- and village-level size estimates. The OD-level correction reduced the household-based proportions at risk (and corresponding CIs) by 50% of the estimated percentage of at-risk householders in the OD who had stayed overnight at another household in the same OD for ≥2 of the past 4 weeks. These parameters are based on World Health Organization guidance for mapping key populations for HIV [9]. The village-level multiplier size estimates were similarly adjusted, based on at-risk householders in the village who had stayed overnight elsewhere in the same village.

All analyses were conducted using multistage survey procedures in Stata (version 15.1; StataCorp). Stata code for all analyses can be found in Multimedia Appendix 2.

### Ethical Considerations

This study was approved by the National Ethics Committee for Health Research of the Ministry of Health of Cambodia (reference 241 NECHR) and the University of California, San Francisco (reference 22-38096). The purpose of the study was explained to all study participants and oral informed consent was obtained. Participants understood that they were free to remove themselves from the study at any time without repercussion. All the data and samples were deidentified and coded toward analysis following institutional review board guidelines.

## Results

### Recruitment

In Phnom Srouch and Sen Monorom ODs, 163 and 138 households, respectively, were enrolled in the survey across the 10 villages selected in each OD. Primary respondents for the households reported data on a total of 844 household members (residents or previous-night visitors) in Phnom Srouch (mean per household 5.2, SD 1.6; range 1-10) and 872 household members in Sen Monorom (mean per household 6.3, SD 3.2; range 1-30). All households approached were enrolled on the first visit attempt. The number of households enrolled per village ranged from 14 to 20 in Phnom Srouch and from 6 to 18 in Sen Monorom.

### Demographics

The mean age of householders was estimated at 29.1 (SE 0.51) years in Phnom Srouch and 26.0 (SE 0.50) years in Sen Monorom (Table 1). Most were aged 18 to 59 years (n=530, 63.4%, in Phnom Srouch and n=480, 56.3%, in Sen Monorom), with a considerable number of children aged 3 to 17 years (from n=249, 29.5% to n=349, 38.5%). More than half of householders were female in Phnom Srouch (n=450, 53%) while 45.7% (n=402) were female in Sen Monorom.

**Table 1:** Estimated demographics of householders aged 3 years and older in the study ODs for this cross-sectional study of forest-exposed populations at high risk of malaria^a^.

			Phnom Srouch (n=844)					Sen Monorom (n=872)		
			n		% (95% CI)		n			% (95% CI)
**Sex**										
	Female	450		53 (50-56)		402			45.7 (41.1-50.5)	
	Male	394		47 (44-50)		470			54.3 (50.0-59.0)	
**Age (years)**										
	3-17	249		29.5 (26.3-32.8)		349			38.5 (34.6-42.6)	
	18-29	239		63.4 (60.0-66.7)		228			56.3 (53.1-59.4)	
	30-59	291		—^b^		252			—	
	≥60	65		7.1 (5.4-9.4)		43			5.2 (3.6-7.4)	
	Mean	—		29.1 (28.1-30.2)		—			26.0 (24.9-27.2)	
	Median	—		25 (24-26)		—			22 (21-23)	

^a^Percentages and CIs are weighted.

^b^Not applicable.

### Household-Based Size Estimates for Populations at Risk

In Sen Monorom, an estimated 706 (79.2%) householders were found to be at risk for malaria as either forest dwellers or forest goers compared to 190 (24.8%) in Phnom Srouch (Table 2). In most villages in Sen Monorom, all participants were forest dwellers, and no village had both forest dwellers and forest goers. In Phnom Srouch, 2 of the selected villages had both forest dweller and forest goers.

**Table 2:** Forest exposure among householders aged 3 years and older for this cross-sectional study of populations at high risk of malaria^a^.

	Phnom Srouch (n=844)			Sen Monorom (n=872)	
	n	% (95% CI)	n		% (95% CI)
				
Forest dweller	132	17.3 (13.9-21.4)	676		77.6 (52.0-91.7)
Forest goer	58	7.5 (5.7-9.9)	30		1.6 (0.2-11.1)
Dweller or goer (“total at risk”)	190	24.8 (21.9-28.1)	706		79.2 (55.5-92.1)

^a^Percentages and CIs are weighted.

Multiplying the proportion at risk by the total OD population led to estimates of 2371 and 20,613 individuals at risk in Phnom Srouch and Sen Monorom, respectively (Table 3).

**Table 3:** District-level population size estimates, based on householder percentage at risk, for 2 operational districts in Cambodia with high malaria prevalence.

District	Population^a^	Survey-estimated percentage at risk (95% CI)^b^	Estimated individuals at risk (95% CI)
Phnom Srouch	9562	24.8 (21.9-28.1)	2371 (2094-2687)
Sen Monorom	26,026	79.2 (55.5-92.1)	20613 (14,444-23,970)

^a^Residents in villages with *Plasmodium falciparum* cases in 2021.

^b^Estimates and CIs are weighted.

The estimates in Table 2 are not adjusted for mobility. Among at-risk participants, 68 (36%) in Phnom Srouch and 280 (32%) in Sen Monorom had stayed overnight at another household in the same OD for ≥2 of the past 4 weeks (Table 4). Reducing the size estimates by 50% of these proportions led to mobility-adjusted estimates of 1945 and 17,315 OD residents at risk, respectively. Notably, the reason reported for 95.8% (387/404) of these stays was “work.”

**Table 4:** District-level population size estimates, adjusted for mobility, for 2 operational districts in Cambodia with high malaria prevalence^a^.

District	At-risk householders meeting mobility criteria		Mobility-adjusted individuals at risk (95% CI)
Phnom Srouch (n=190)	68	36 (24.5-45.5)	1945 (1717-2203)
Sen Monorom (n=706)	280	32 (19.9-47.2)	17,315 (12,133-20,135)

^a^Estimates and CIs are weighted.

### Multiplier Size Estimates

Of the 9 villages where BITE forest packs were distributed, 4 were subsequently randomly selected for the survey (Table 5). All participants in these villages were forest dwellers, based on their survey responses. Between 67% (n=59) to 98.8% (n=82) of the at-risk population across these villages were estimated to have received the forest packs prior to the survey.

**Table 5:** Village-level population size estimates, based on the multiplier method, for villages in Cambodia with high malaria prevalence^a^.

Village	Resident population	Survey participants classified as at risk	At-risk survey participants who received BITE tools			Individuals given BITE tools^b^ as per distribution records (*M*)		Estimated individuals at risk (95% CI)
			n	% (95% CI); *p*				
Chak Cha	1500	82/82	0	—^c^	160		—	
Pu Char	329	88/88	59	67 (66.8-67.3)	107		160 (129-190)	
K uon	130	83/83	70	84.3 (84.0-84.6)	164^d^		195 (165-224)	
Kdaoy	275	83/83	82	98.8 (98.7-98.8)	160		162 (137-187)	

^a^Percentages and CIs are weighted estimates_._

^b^Approximated as ¼ (BITE [bite interruption toward elimination] packs distributed).

^c^Not applicable.

^d^This figure, gathered from distribution records, is greater than the population size of the village, which was based on the available village registry. Table 5 includes villages included in both the survey and program implementation.

Combining these proportions with the total populations of the villages, we estimate 160, 195, and 162 residents at risk, respectively (Table 5). Multiplier estimates could not be calculated for Chak Cha village because no survey participants there had received the BITE packs.

The estimated percentage of householders who met the within-village mobility criterion varied widely, from 6% (5/83) in Kdaoy to 43.2% (38/88) in Pu Char (Table 6). The mobility adjustment reduced the size estimates in the 3 villages to 125, 186, and 157 individuals at risk, respectively.

**Table 6:** Village-level population size estimates, adjusted for mobility for villages in Cambodia with high malaria prevalence.

Village	Householder meeting mobility criteria		Mobility-adjusted individuals at risk (95% CI)	Uncertainty interval^a^
	n	% (95% CI)		
Chak Cha	12/82	14.6 (14.4-14.8)	—^b^	—
Pu Char	38/88	43.2 (42.7-43.6)	125 (101-149)	107-149
K uon	7/83	8.4 (8.2-8.7)	186 (158-215)	cannot be determined^c^
Kdaoy	5/83	6 (5.9-6.2)	157 (133-181)	160-181

^a^The uncertainty interval goes from the number of individuals who received BITE (bite interruption toward elimination) tools to the population size of the village.

^b^Not applicable.

^c^The upper limit of the uncertainty interval is meant to be the total population size of the village; however, BITE pack distribution data indicates that more packs were distributed than there are individuals in the village.

However, the 95% CI limits of the multiplier estimates (before and after the mobility adjustment) were greater than the total number of residents in K uon and Kdaoy villages. Furthermore, in all 3 villages, the CI’s lower limit was less than our approximation of the number of people who received forest packs. We therefore report an additional “uncertainty interval” bounded by the number of BITE pack recipients and the village population (Table 6).

## Discussion

### Overview

Based on a survey of forest-exposed individuals in 2 high malaria transmission ODs in Cambodia, this study estimates that there are 1945 individuals in Phnom Srouch OD and 17,315 individuals in Sen Monorom OD who may be at high risk for malaria based on their proximity or travel to forested areas. A higher proportion of people in Sen Monorom (nearly 80%) were classified as at risk compared to Phnom Srouch (approximately 25%), which is likely a result of decreasing forest stands in Kampong Speu province [10]. These estimates of the population at risk can provide essential data to plan and budget for government or nongovernmental organization–led anti-malaria campaigns and support the targeting of health programs to those likely to be at greatest risk.

As countries in the GMS continue to move toward malaria elimination, the populations at risk of malaria have become smaller and are increasingly those exposed to forests [3,11,12]. Unlike in high-burden settings, where vector-control centers on mass distribution or campaigns over large areas and populations, in low-burden settings it becomes increasingly important to target and tailor prevention to the diminishing population of individuals at continued risk. Intervention targeting may also require alternative delivery methods to access hard-to-reach populations [13]. To efficiently target surveillance, prevention, and control activities, national malaria programs require accurate estimates of the size and locations of the subgroups at risk. This study demonstrated a straightforward approach using household surveys, which is likely to be feasible in contexts where national malaria programs can enlist the support of local health facility staff or village or mobile malaria workers, and where most individuals at risk can be enrolled via their households. In contrast to recent applications of the multiple source capture-recapture method for population size estimation in Lao People's Democratic Republic [14] and Namibia [13], which have relied on serial household surveys and multiple sources of program data, the methods described here require fewer data sources, fewer contacts with participants, and less complicated statistical analysis.

Findings in this study indicate that, of the 2 ODs, which were among the high-risk districts in Cambodia, a much higher proportion of at-risk individuals, as defined in this study, was estimated in Sen Monorom OD in Mondulkiri province compared to Phnom Srouch OD in Kampong Speu province. We speculate that this difference is largely driven by 2 factors: the amount of intact forest remaining in Sen Monorom OD and the amount of travel to the forest from the OD. In fact, the former factor directly influences the latter; when there is more forest available, people are more likely to travel to the forest for purposes of logging, hunting, collection of nontimber forest products, farming, and other activities. In contrast to Mondulkiri, forested areas in Kampong Speu are becoming increasingly rare [10], with fewer people making a living in forest-related occupations.

The study was conducted in April, after the end of the malaria season, which typically runs from August to December or early January. The malaria season coincides with the rainy season when people are likely to be travelling to the forest. While people may have stopped going to the forest as frequently by the time this study was conducted, the survey used was able to capture any travel to the forest within the last 3 months, which should identify those individuals who typically travel to the forest during the rainy season. However, people who may have traveled to one of the ODs in the study from another location temporarily (ie, migrant workers, migrant populations, seasonal workers) may have been missed by this survey if they returned to their homes between the end of the rainy season and when the study began.

It is helpful, for purposes of malaria elimination, to have multiple size estimation tools, because there are limitations to any single method [15]. Multiple strategies also allow countries greater flexibility to select the best approach for a given context. However, the size estimates generated here using the multiplier method—which could only be undertaken in the 4 villages surveyed where BITE tools had been distributed—produced, in part, unrealistic results. Although all survey participants in these villages were forest dwellers, which suggests that the entire village population was proximate to or inside the forest, the multiplier estimates of 160 (95% CI 129-190) individuals at risk in Pu Char village and of 162 (95% CI 137-187) in Kdaoy village were far below the resident populations of 329 and 275 people, respectively. This level of underestimation seems too great to be due to children aged 3 years older in the population counts, who were not included in the surveys. Instead, the large error is likely due to the fact that the number of unique individuals who received forest packs needed to be approximated by assuming the same individuals were reached during each round of distribution. Conversely, the multiplier estimate of 195 (95% CI 165-224) individuals at risk in K uon village is larger than the total village population of 130 residents, which may be due to underestimation of the parameter *p* (the survey percentage), potentially due to underreporting. The K uon overestimate persisted even after accounting for householders who frequently stayed at other households in the same village.

This study was based on a probability survey of households so that it can be considered representative of the household population in each OD. Since respondents were successfully enrolled from all selected households, with no refusals, there was no potential for bias due to differential rates of participation in the study. The study team attributes this to close coordination with village leaders and the presence of VMWs on the field team. Furthermore, there is likely to be minimal error owing to residents’ being away from the selected households at the time of the survey since data were collected on all household members and recent visitors.

While this study had the benefit of being part of the larger Project BITE research program, which included providing forest packs to some villages independent of this population size estimation study, there are other approaches to leveraging existing malaria service delivery or surveillance activities to enable more accurate estimates using the multiplier method. For example, if a population size estimate study is being conducted by interviewing individuals who arrive at a public health clinic, the proportion of interviewed individuals that received chemoprophylaxis from that clinic or local VMWs might be used as a multiplier to further refine estimates. There are numerous such ways that a sample population can be subdivided based on receiving or enrolling in another service in order to create more accurate estimates.

When conducting future population size estimate surveys, it is recommended to first engage in relationship building with target villages and populations, especially if the survey is enquiring about potentially elicit or illegal activities (as was the case in this study, where illegal logging is common among the target population). Creating a relationship based on mutual trust will allow for greater honesty in survey responses, and thereby more accurate estimates. Furthermore, it is important to reach those households or individuals who were randomly selected, and if necessary multiple attempts should be made. Especially in a study like this, where mobility and exposure to forests is central to the population being studied, care must be taken that selected individuals are not skipped if not immediately available, as it is likely that the person is unavailable precisely because they are part of the population being studied. The study team must be flexible with where and when they are on-site to conduct interviews and work with village leaders to communicate the needs and enrolment criteria of the study, in order to achieve maximum representation from the randomly selected group.

### Limitations

Our findings are subject to important limitations. The size estimates do not capture any individuals who resided entirely at work sites (in forested areas or otherwise) and did not belong to a household in the village, such as foreign workers. Future studies may improve upon this by working with local village chiefs and other leaders to identify these groups and include them in the sampling frame of households. The mobility adjustment prevented double counting of individuals who may live between 2 households, but not 3 or more. There may also be a small degree of error due to householders’ not knowing whether their households were located within 1 km of the forest; yet, in only 16 of the 301 households surveyed (5%) was there disagreement among participants on this measure and resolving it (by assuming the majority response was correct) changed our estimate of the prevalence of forest dwellers by less than 1%. The size estimates also do not include anyone living away from the forest who traveled there less than weekly, or who began doing so subsequent to the survey (ie, after mid-April). The OD-level size estimates—which were based on percentages of householders—may have been underestimated if the primary respondents (heads of household or other adults present at the time) were unaware of others’ forest-going activity. However, in this setting of small villages, the study team believes this is unlikely as most family and community members are aware of the activities of others. A source of overestimation was that available population counts which were used to multiply the percentages at risk estimated from the survey could not be subset by age, whereas survey data could only be collected on individuals older than age 3 due to concerns raised during ethical review of the study protocol. For a more aligned calculation, future surveys should collect data on the ages of all householders (regardless of whether they are considered study-eligible) in order to estimate the age distribution in the general population, if unavailable from census data.

A multiplier estimate for Chak Cha village could not be calculated because none of the survey participants had received the forest pack (leading to a *p* of 0); this was due to low coverage of the tools in the village (about 7%, according to program data). To avoid this situation, the authors recommend using a publicly available sample size calculator [6] to determine the size of the multiplier required when planning for multiplier size estimates. It is also important to note that the “services multiplier method”—commonly used in HIV epidemiology [6,7]—works best when program data accurately reflect the number of unique individuals who have accessed a specific service in the study area during a specific time period. Recent adaptations of the capture-recapture method have been introduced that do not require program data [16,17].

Finally, we note that the precision of the size estimates for Sen Monorom (where villages were randomly sampled) was far less than in Phnom Srouch (where all villages with recent *P falciparum* cases were surveyed) due to the considerable degree of village-level variation in our risk measures and the relatively small number of households selected per village, owing to budget limitations. To improve precision, future studies should aim to collect data from a larger number of households and ensure this target is met even after the planned sample of individuals has already been achieved.

### Conclusions

This study provides a robust and replicable method for estimating the number of individuals at high risk of malaria due to forest exposure in the last remaining pockets of malaria transmission in Cambodia and across the GMS. With perpetually constrained budgets for malaria elimination activities, having a reliable understanding of the estimated size of the population at risk for malaria allows national malaria programs to appropriately plan for and implement critical prevention and surveillance activities.

## Appendix

Multimedia Appendix 1 Survey questionnaire.

Multimedia Appendix 2 Data analysis STATA code.

## Abbreviations

BITE: bite interruption toward elimination
GMS: Greater Mekong Subregion
MIS: malaria information system
OD: operational district
VMW: village malaria worker
