# Supplementary material for: Estimating the Size of Populations at High Risk of Malaria in 2 Operational Districts in Cambodia: Household-Based Survey
Source: JMIR Public Health Surveill. 2024 Sep 27;10:e58584. doi: 10.2196/58584 (PMC11470217; doi:10.2196/58584)
Supplement: Multimedia Appendix 1 [file publichealth_v10i1e58584_app1.docx]

| **Section 1: Household Identiﬁcation** |
| --- |
| Date____/_____/_______ |
| 1. Supervisor Number [_____________________] |
| 2. OD Code [_____________________] |
| 3. Village Code [_____________________] |
| 4. Household ID [_____________________] |
| Household ID [_____________________] |
| 1. Participant ID [_____________________] |
| 1. Does this individual meet the inclusion criteria for the study? If No, STOP   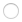 No  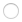 Yes |
| 1. Has this individual read and understood the informed consent form, and agree to participate in the study? If No, STOP   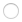 No  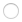 Yes |
| 1. What visit attempt is this? [_____________________] |
| **INSTRUCTIONS TO INTERVIEWER**  Participants should provide information for all residents and visitors aged 3 and older, whether or not they are at home at the time of the visit, and whether or not they are part of the same family.  A resident is anyone who regularly sleeps at this household.  A visitor is anyone else who slept at this household last night.  The participant should answer on behalf of all residents and visitors, to the best of his or her ability. However, the participant should feel free to confer with them in order to provide a more accurate response.  Up to 3 visit attempts will be made for selected households, before selecting a replacement household.  Please tell me the names of all people who usually live in this household (in any structure), and anyone else who slept here last night. But only those who are aged 3 or older. To protect conﬁdentiality, I will write down only the ﬁrst TWO letters of each person's given name. If two people here have the same ﬁrst two letters, I will write down the ﬁrst THREE letters. For example, instead of "Chea", I would write "CH". This will be the person's nickname for all of the questions. |
| **Section 2: Household Listing**  Now we would like to ask you some information about time spent at this household and at other places in the past 4 weeks.  We would ﬁrst like to ask you some information about the members of this household, including anyone who usually sleeps here and any recent visitors. |
| Q1. Individual # [_____________________] |
| Q2. Nickname (ﬁrst 2/3 letters of given name) [_____________________] |
| Q3Y. Age (years) [_____________________] |
| Q3M. Age (months) [_____________________] |
| **Q4. What is the relationship of (NICKNAME) to the head of household?**  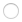 Head of household  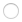 Spouse (wife/husband/partner)  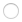 Son or daughter  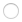 Son-in-law or daughter-in-law  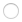 Adopted/foster/stepchild  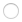 Grandchild  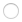 Parent  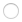 Aunt or uncle  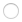 Grandparent  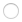 Other relative  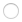 Not related (friend, vistor) |
| Q5. Is (NICKNAME) male or female 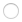 Male  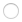 Female |
| **Q6. In the past 4 weeks, did (NICKNAME) stay overnight any place else away from this household?**  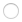 No  Yes |
| Q7. If (NICKNAME) stayed overnight outside this household in the past 4 weeks, what was the primary reason? 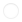 Work  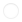 Away at school / studying  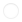 Staying with relatives or friends  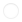 Holidays / travel for leisure  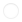 Hospitalized or taking care of patient  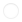 Don't know  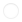 Other  **Describe other**  [___________________________________________________________________________________] |
| Q8. Is the other place where (NICKNAME) stayed overnight… in this village, in another village in this district, or outside this district? 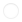 This village  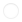 Another village in this district  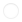 Outside of district  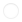 Don't know |
| **Q9. In the past 4 weeks, approximately how many weeks did (NICKNAME) spend at this other place?**  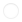 Less than one week  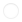 1 week  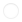 2 weeks  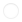 3 weeks  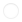 4 weeks  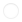 Don't know |
| Q10. In the past 4 weeks, approximately how many weeks did (NICKNAME) spend the night here at this household? 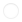 Less than one week  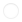 1 week  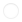 2 weeks  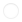 3 weeks  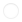 4 weeks  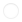 Don't know |
| Is this household inside the forest or within 1km of the forest? 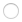 No  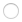 Yes |
| Q11. During the dry season so far this year up to today, did (NICKNAME) spend any time in the forest? 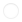 No  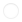 Yes  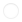 Don't know |
| **Q12. During the dry season so far this year up to today, did (NICKNAME) ever go to the forest at least 1 day per week?**  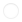 No  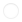 Yes  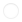 Don't know |
| **Q13. In which months of this dry season did (NICKNAME) go to the forest at least 1 day per week? Select all that apply**  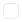 January  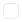 February  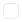 March  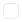 April  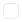 Don't know |
| **Section 3: Participation in BITE Activities** |
| **Did this village receive products from BITE?**  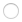 No  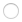 Yes  We would now like to ask you about some recent mosquito bite prevention activities in this district. |
| **Q14. Between October and January, did (NICKNAME) provide a blood sample to Project BITE?**  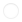 No  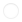 Yes  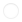 Don't know |
| **Q15. Between October and January, did (NICKNAME) receive any items to help prevent mosquito bites from a VMW as part of Project BITE?**  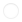 No  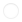 Yes  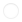 Don't know |
| **Q16. What kinds of items for mosquito bite prevention did (NICKNAME) receive from a VMW?**  *Wait for participant respond spontaneously. Do not read the options.*  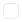 Topical Repellent  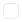 Spatial Repellent  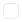 Etofenprox treatment for clothing  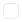 None of the above  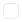 Don't know  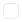 Other  **Describe other** [___________________________________________________________________________________] |
