# Supplementary material for: Estimating the Size of Populations at High Risk of Malaria in 2 Operational Districts in Cambodia: Household-Based Survey
Source: JMIR Public Health Surveill. 2024 Sep 27;10:e58584. doi: 10.2196/58584 (PMC11470217; doi:10.2196/58584)
Supplement: Multimedia Appendix 2 [file publichealth_v10i1e58584_app2.docx]

* Cambodia PSE for forest workers using HH survey and multipliers

#delim ;

set more off;

* folders;

local dir "~/Documents/Projects/Projects-working/UCSF/Cambodia PSE/Analysis";

local datadir = "`dir'/../Data";

local data_prelim = "`datadir'/PSE Full Dataset 18apr23.xlsx";

local data_final = "`datadir'/PSE Full Dataset cleaned updated 01jul23.xlsx";

local sheet = "data_fmtd";

local dataout = "pse cambodia";

cd "`dir'";

* import final excel data;

import excel using "`data_final'", sheet("`sheet'") cellrange(A2)

firstrow case(lower) clear;

compress;

save "`dataout'", replace;

*/;

* reload saved data;

capture log close;

log using "pse_cambodia.log", replace;

use "`dataout'", clear;

do labels;

* dates;

* "date" reflects end date, which differs from start date;

* some dates are missing;

* tabulate start and end dates;

gen start_mo = substr(start, 6, 2);

gen start_day = substr(start, 9, 2);

destring start_mo start_day, replace force;

gen end_mo = substr(end, 6, 2);

gen end_day = substr(end, 9, 2);

destring end_mo end_day, replace force;

ren start start_old;

ren end end_old;

gen start = mdy(start_mo, start_day, 2023);

gen end = mdy(end_mo, end_day, 2023);

format start end %td;

lab var start "start date of survey, from machine recorded start";

lab var end "end date of survey, from machine recorded end";

drop start_mo start_day end_mo end_day;

tab start;

tab end;

ren date date_old;

lab var date_old "end date as reported, but some missing";

* svyset for Phnom Sruouch (od==1);

*svyset hhuic [pw=weight], strata(vcode) fpc(fpc_village) vce(linearized);

* svyset for Senmonorom (od==2);

*svyset vcode [pw=weight], fpc(fpc_od) vce(linearized) || hhuic;

* IDENTIFIERS;

lab def odl 1 "Phnom Srouch" 2 "Senmonorom";

lab val od odl;

* hh UIC;

* there should be 301 HHs, as in the HH-level spreadsheet;

quietly duplicates report od vcode hhid2;

return list;

gen hhuic = string(od) + string(vcode) + string(hhid2);

lab var hhuic "Household UIC";

/* Eligibility;

* Dave confirmed that all participants were eligible and consented,

* that "no" values in data reflect data issue

*/;

tab elig, m;

tab consent, m;

summ ageyr,d;

* TRIM STRINGS;

foreach x of var village_name hhinforest goforest_any goforest_1daywk

bite_bloodsample bite_self bite_gotitems bite_top bite_spat bite_etof

eligible consented nickname

relation sex overnight overnight_rsn overnight_loc overnight_time*

{;

replace `x' = trim(`x');

};

* SAMPLING WEIGHTS;

* weights are calculated in "Sampling plan & weights PSE Cambodia 15jun23.xlsx";

gen weight=.;

* Phnom Srouch;

replace weight=1.71 if vcode==1 & od==1;

replace weight=2.24 if vcode==2 & od==1;

replace weight=1.51 if vcode==3 & od==1;

replace weight=2.01 if vcode==4 & od==1;

replace weight=1.71 if vcode==5 & od==1;

replace weight=1 if vcode==6 & od==1;

replace weight=1.01 if vcode==7 & od==1;

replace weight=1.55 if vcode==8 & od==1;

replace weight=1.5 if vcode==9 & od==1;

replace weight=1 if vcode==10 & od==1;

* Mondulkiri / Senmonorom;

replace weight=2.577 if vcode==5 & od==2;

replace weight=37.413 if vcode==11 & od==2;

replace weight=13.402 if vcode==17 & od==2;

replace weight=7.211 if vcode==24 & od==2;

replace weight=3.301 if vcode==28 & od==2;

replace weight=12.541 if vcode==30 & od==2;

replace weight=2.49 if vcode==33 & od==2;

replace weight=1.868 if vcode==35 & od==2;

replace weight=2.491 if vcode==37 & od==2;

replace weight=1 if vcode==38 & od==2;

assert weight!=.;

by od, sort: table vcode, c(min weight max weight);

* un-normalize weights, to see if it improves CIs;

replace weight = weight * 9.4 if od==1;

replace weight = weight * 4.3 if od==2;

* Finite population corrections (FPCs);

* OD-level FPCs;

gen fpc_od=.;

replace fpc_od=9562 if od==1;

replace fpc_od=26026 if od==2;

lab var fpc_od "#residents in OD in villages with pf cases in 2022";

assert fpc_od!=.;

* village-level FPCs;

* list participants per village, to calculate village-level FPC in Excel;

by od, sort: tab vcode od;

gen fpc_village=.;

replace fpc_village=1214 if vcode==1 & od==1;

replace fpc_village=1266 if vcode==2 & od==1;

replace fpc_village=1041 if vcode==3 & od==1;

replace fpc_village=1277 if vcode==4 & od==1;

replace fpc_village=1107 if vcode==5 & od==1;

replace fpc_village=670 if vcode==6 & od==1;

replace fpc_village=608 if vcode==7 & od==1;

replace fpc_village=838 if vcode==8 & od==1;

replace fpc_village=899 if vcode==9 & od==1;

replace fpc_village=642 if vcode==10 & od==1;

replace fpc_village=669 if vcode==5 & od==2;

replace fpc_village=981 if vcode==11 & od==2;

replace fpc_village=553 if vcode==17 & od==2;

replace fpc_village=321 if vcode==24 & od==2;

replace fpc_village=264 if vcode==28 & od==2;

replace fpc_village=1240 if vcode==30 & od==2;

replace fpc_village=210 if vcode==33 & od==2;

replace fpc_village=329 if vcode==35 & od==2;

replace fpc_village=130 if vcode==37 & od==2;

replace fpc_village=275 if vcode==38 & od==2;

lab var fpc_village "#residents in village";

assert fpc_village!=.;

* DEMOGRAPHICS;

gen male=sex=="male";

gen agecat = cond(ageyr<=17, 1,

cond(ageyr<=59, 2,

cond(ageyr>=60 & ageyr!=., 3, .)));

lab def agel 1 "3-17" 2 "18-59" 3 ">=60";

lab values agecat agel;

tab od sex, row m;

tab od agecat, row m;

preserve;

keep if od==1;

svyset hhuic [pw=weight], strata(vcode) fpc(fpc_village) vce(linearized);

svy: tab sex, ci;

svy: tab agecat, ci;

svy: mean ageyr;

epctile ageyr, p(50) svy;

restore;

preserve;

keep if od==2;

svyset vcode [pw=weight], fpc(fpc_od) vce(linearized) || hhuic;

svy: tab sex, ci;

svy: tab agecat, ci;

svy: mean ageyr;

epctile ageyr, p(50) svy;

restore;

* RISK CRITERIA

* forest dweller;

replace hhinforest = trim(hhinforest);

assert inlist(hhinforest, "yes", "no");

gen dweller = hhinforest=="yes";

tab od dweller, row m;

lab var dweller "forest dweller";

* check that hhinforest is same for all participants in hh;

sort hhuic participantid;

l od vcode hhuic hhinforest participantid dweller if dweller [_n] != dweller [_n-1] & hhuic [_n]==hhuic [_n-1], sepby(hhuic) noobs;

assert dweller [_n] == dweller [_n-1] if hhuic [_n]==hhuic [_n-1];

* forest goer;

tab goforest_any goforest_1day if !dweller,m;

gen goer = goforest_1day=="yes";

replace goer=. if goforest_any=="";

replace goer=0 if dweller==1;

assert goer!=1 if dweller==1;

tab goer if !dweller,m;

lab var goer "forest goer";

* at risk: either dweller or goer;

* (dweller has no missings);

gen atrisk = dweller==1 | goer==1 if goer!=.;

lab var atrisk "forest dweller or goer";

tabstat dweller goer atrisk, s(n sum mean) by(od) notot;

* Unweighted risk proportions;

by od, sort: ci proportion dweller goer atrisk;

*** Weighted risk proportions - weighting scheme differs by OD ***;

* (svyset interprets fpc as N (pop size) if fpc>1);

* Phnom Srouch;

* villages are strata since all were included;

preserve;

keep if od==1;

* ESTIMATES USING UNSTANDARDIZED WEIGHTS - PHNOM SROUCH;

svyset hhuic [pw=weight], strata(vcode) fpc(fpc_village) vce(linearized);

svy: proportion dweller goer atrisk;

* ESTIMATES USING STANDARDIZED WEIGHTS - PHNOM SROUCH;

qui summ weight;

gen sweight = (weight - r(mean)) / r(sd);

*svyset hhuic [pw=sweight], strata(vcode) fpc(fpc_village) vce(linearized);

*svy: proportion dweller goer atrisk;

di "STANDARDIZED SAMPLING WEIGHTS for Phnom Srouch";

summ sweight, d;

restore;

* Senmonorom;

* villages are stage 1 since they were sampled;

preserve;

keep if od==2;

* ESTIMATES USING UNSTANDARDIZED WEIGHTS - SENMONOROM;

svyset vcode [pw=weight], fpc(fpc_od) vce(linearized) || hhuic;

svy: proportion dweller goer atrisk;

* ESTIMATES USING STANDARDIZED WEIGHTS - SENMONOROM;

qui summ weight;

gen sweight = (weight - r(mean)) / r(sd);

*svyset vcode [pw=sweight], fpc(fpc_od) vce(linearized) || hhuic;

*svy: proportion dweller goer atrisk;

di "SAMPLING WEIGHTS for Senmonorom";

summ sweight, d;

restore;

* Village-level variation in risk estimates (unweighted);

* the cause of the wide CIs;

by od, sort: table vcode, c(mean dweller mean goer);

/*** MOBILITY CORRECTION FACTORS FOR DISTRICTS ****

*

* survey participants were:

* residents, anyone who regularly sleeps at this HH

* visitors, anyone else who slept at this HH last night

*/;

* In the past 4 weeks, did any at-risk participants stay overnight ;

* in other villages included in the study?;

tab od overnight if atrisk, m;

* a lot of mobility within village and district;

by od, sort: tab overnight overnight_location if atrisk,m;

* were they gone long enough to risk getting counted ;

* as a "usual resident" in another HH in the study area?;

* 18% of those who traveled spent >=2 weeks in the past 4 weeks there;

* 48% spent >=1 week;

tab overnight_time_there if atrisk & overnight=="yes";

tab overnight_time_here overnight_time_there if atrisk & overnight=="yes";

* Mobility identifier for districts;

assert inlist(overnight, "yes", "no");

gen mobile_district = inlist(overnight_location,

"another_village_in_this_district",

"this_village");

tab od mobile_district if atrisk, row m;

* Phnom Srouch;

preserve;

keep if od==1;

svyset hhuic [pw=weight], strata(vcode) fpc(fpc_village) vce(linearized);

svy, subpop(if atrisk): proportion mobile_district;

restore;

* Senmonorom;

preserve;

keep if od==2;

svyset vcode [pw=weight], fpc(fpc_od) vce(linearized) || hhuic;

svy, subpop(if atrisk): proportion mobile_district;

restore;

* Most stayed overnight elsewhere for WORK;

tab overnight_rsn if mobile_district==1;

*** Mobility correction factor for villages ***;

* Mobility identifier for districts;

gen mobile_village = overnight_location=="this_village";

tab od mobile_district if atrisk, row m;

* MULTIPLIER ESTIMATES;

* whether village got BITE - version corrected by Dyna;

* 0/1/99. 99s represent villages where BITE was distributed just once (instead of 4 times);

* exclude 99s from the multiplier estimates;

* exclude Phnom Srouch (Kampong Speu province) from the multiplier estimates;

assert inlist(bite_corrected, 0, 1, 99);

recode bite_corrected (99=0), gen(bite_village);

replace bite_village=0 if od==1;

lab var bite_village "Village in Senmonorom OD and received 4 rounds of BITE distribution";

assert inlist(bite_village, 0, 1);

* check whether same within village (confirmed);

tab vcode bite_village,m;

table vcode, c(min bite_village max bite_village);

tab village_name if bite_village==1;

* area unique identifier (sub-annex or main village);

gen area = string(vcode) + " " + village_name;

* Total N and #at-risk in areas;

table area if bite_village==1, c(count vcode sum dweller sum goer sum atrisk);

* received any BITE items?;

* check consistency of responses;

tab bite_got bite_topical,m;

tab bite_got bite_spatial,m;

tab bite_got bite_etof,m;

tab bite_ot,m;

tab bite_oth_specify,m;

assert inlist(bite_got, "yes", "no", "n/a");

foreach x in "topical" "spatial" "etof"

{;

assert inlist(bite_`x', "0", "1", "n/a");

gen `x' = bite_`x'=="1" if inlist(bite_`x', "0", "1");

replace `x'=0 if bite_got=="no";

* respondent said their village didn't get BITE, so they weren't asked

* if they personally got BITE items (recorded as 'n/a');

* recode as 0 so they are included in the denominator;

replace `x'=0 if bite_village==1 & bite_selfreport=="no" & bite_got=="n/a";

lab var `x' "received `x' during period";

};

gen bite_any = (topical==1 | spatial==1 | etof==1) if topical!=.;

lab var bite_any "received a BITE tool during period";

* got any BITE items, all areas in survey;

by od, sort: summ topical spatial etof bite_any;

* got any BITE items, multiplier areas only;

summ topical spatial etof bite_any if bite_village;

* # participants who received BITE tools;

tab area bite_any if bite_village==1 & atrisk==1;

* calculate p(received BITE) w/CI by village, Senmonorom OD only;

keep if od==2;

svyset vcode [pw=weight], fpc(fpc_od) vce(linearized) || hhuic;

svy, subpop(if bite_village==1 & atrisk==1): proportion bite_any;

foreach a in "30 Chak Cha Village"

"35 Pu Char"

"37 K uon"

"38 Kdaoy"

{;

di " ";

di "AREA: `a'";

di " ";

svy, subpop(if bite_village==1 & area=="`a'"): proportion bite_any;

* mobility correction factor for `a';

tab mobile_village if area=="`a'";

svy, subpop(if area=="`a'"): proportion mobile_village;

* unadjusted estimate for `a' to calculate DEFF;

ci prop bite_any if bite_village==1 & area=="`a'";};
